# Age-related Behavioural Change in Cornelia de Lange and Cri du Chat Syndromes: A Seven Year Follow-up Study

**DOI:** 10.1007/s10803-019-03966-6

**Published:** 2019-04-02

**Authors:** Lisa Cochran, Alice Welham, Chris Oliver, Adam Arshad, Joanna F. Moss

**Affiliations:** 1 Cerebra Centre for Neurodevelopmental Disorders, School of Psychology, University of Birmingham, Birmingham, UK; 2 Institute of Cognitive Neuroscience, University College London, London, UK; 3 University of Leicester, Leicester, UK; 4 College of Medical and Dental Sciences, University of Birmingham, Birmingham, UK

**Keywords:** Cornelia de Lange syndrome, Cri du Chat syndrome, Autism spectrum disorder, Behaviour, Age

## Abstract

Age-related behavioural change in Cornelia de Lange syndrome is poorly understood. We report a 7 year follow-up study of adaptive behaviour, autism spectrum disorder symptomatology, language skills and behavioural characteristics in 30 individuals with Cornelia de Lange syndrome, compared with 18 individuals with Cri du Chat syndrome. The proportion of individuals with Cornelia de Lange syndrome meeting criteria for autism spectrum disorder on the Autism Diagnostic Observation Schedule increased, although patterns of change were complex. For both syndrome groups, absolute levels of adaptive ability were stable and receptive language improved, suggesting that changes over time do not result from an overall decline in ability. Reliable change index scores indicate heterogeneity within both groups in the occurrence of improvement or decline.

## Introduction

Cornelia de Lange syndrome (CdLS) is a rare multisystem genetic disorder that affects approximately one child in every 40,000–100,000 (O’Brien and Yule 1995). The syndrome is associated with unusual facial features, limb malformations (Selicorni et al. 2007) and a wide range of health conditions (Hall et al. 2008). Associated intellectual disability (ID) is typically within the severe to profound range, although a proportion of individuals may have moderate or mild ID (Sloneem et al. 2009).

The most common known genetic cause of CdLS is mutation of the NIPBL gene, which accounts for up to 80% of cases (Krantz et al. 2004; Tonkin et al. 2004; Huisman et al. 2013). Mosaicism for a NIPBL mutation is identified in 23% of individuals (Huisman et al. 2013). A number of other less common causal mutations have also been identified. Mutations in SMC1a and SMC3 have been found to account for CdLS in a further 5% of affected individuals (Deardorff et al. 2007; Musio et al. 2006), and more recently, mutations in the HDAC8 and RAD21 genes have been identified in a small number of cases (Deardorff et al. 2012). All of these genes encode proteins related to the cohesin complex pathway which is thought to be central to regulating and maintaining DNA production.

Behavioral characteristics of CdLS include social avoidance, repetitive and self-injurious behaviors and hyperactivity (Berney et al. 1999; Hyman et al. 2002; Moss et al. 2009; Oliver et al. 2008). Autism spectrum disorder (ASD) characteristics are common, and may be extensive enough to warrant diagnosis of an ASD in 51% − 67% of individuals (Oliver et al. 2008; Basile et al. 2007; Bhuiyan et al. 2006; Moss et al. 2008; Oliver et al. 2011; Srivastava et al. 2014; Nakanishi et al. 2012). There is emerging evidence indicating broad age related changes in CdLS including increased anxiety, social withdrawal, low mood, insistence on sameness and challenging behavior (Berney et al. 1999; Moss et al. 2017; Nelson et al. 2014; Oliver et al. 2011; Sarimski et al. 1997). Reid et al. (2017) also report significantly greater impairments in executive function skills with increasing chronological age and Kline et al. (2007) describe an early onset of the physical signs of ageing in this population. To date, no studies have evaluated the potential for change in broader aspects of functioning such as adaptive behaviour and language skills.

It has been postulated that the causal mutations in the cohesion pathway associated with CdLS might account for the reported changes with age in the syndrome. Compromised function of the cohesin pathway (resulting from the genetic mutations which cause CdLS) has been implicated in these behavioural and cognitive changes in CdLS, due to the role of this pathway in neural maintenance and repair (Kline et al. 2007). Evidence also indicates down-regulation of proteins involved in the response to oxidative stress and an increase in global oxidative stress in CdLS cell lines which may be directly linked to the phenotypic changes in the syndrome (Gimigliano et al. 2012). While this requires further investigation in CdLS, the role of oxidative stress in neurodegeneration has been evidenced in other disorders including Alzheimer’s disease.

To date, the majority of research reporting changes with age in CdLS has relied upon cross-sectional data or short term follow up studies conducted over a 2 year period (Cochran et al. 2015; Nelson et al. 2014). In the current study, we evaluate adaptive behaviour, language skills and behavioural characteristics (ASD symptomatology, mood and repetitive behaviour) over a 7 year follow-up period in 30 individuals with CdLS and a contrast group of individuals with Cri du Chat syndrome (CdCS).

CdCS is associated with similar levels of severe/profound ID, social communication deficits and stereotyped behavior to those reported in individuals with CdLS, making it a suitable contrast group for individuals with CdLS. CdCS affects approximately one in 50,000 births (Dykens et al. 2000) and results from a deletion of chromatin from the short arm of chromosome five. The deletion is present in 85% of cases; 10–15% are familial with more than 90% due to a parental translocation and 5% due to an inversion of chromosome five (Van Buggenhout et al. 2000). Behaviours associated with CdCS include self-stimulatory/repetitive behaviors, self-injurious behavior, aggression, temper tantrums, hyperactivity, poor concentration/distractibility and impulsivity (Collins and Cornish 2002; Cornish et al. 1998; Cornish and Pigram 1996; Clarke and Dykens 1997; Dykens et al. 2000 and; Sarimski 2003). Several studies report social interaction skills as being a relative strength of individuals with CdCS (Carlin 1990; Cornish and Pigram 1996). Very little is known about the developmental profile of behaviors in CdCS and the outcome for adults with the syndrome.

The primary aim of the current study was to conduct a 7 year follow up of individuals with CdLS and CdCS. We report on progress and changes made in both groups over this period in adaptive behaviour and language skills and in behavioural characteristics including ASD symptomatology, mood and repetitive behaviour. A secondary aim was to consider patterns of change over time in these domains for individuals.

## Method

### Design

This study is a 6.5–7.5 year longitudinal follow-up of behavioural, cognitive and linguistic abilities in CdLS and CdCS. Each participant was initially assessed as part of a previous study conducted at Cerebra Centre for Neurodevelopmental Disorders, University of Birmingham, UK between 2004 and 2005 (Time 1; T1). In this study, outcomes 6.5–7.5 years later (Time 2; T2) in the areas of behaviour, language ability and adaptive behaviour skills were investigated.

### Recruitment

Parents and carers of individuals with CdLS and CdCS and their children were invited to participate in this follow-up study. Thirty-two individuals with CdLS and 21 individuals with CdCS and their carers who participated in a research study between 2004 and 2005 at Cerebra Centre for Neurodevelopmental Disorders, University of Birmingham, UK were invited to take part in the current study. At T1, participants were recruited through a database at Cerebra Centre for Neurodevelopmental Disorders, University of Birmingham, UK of individuals with CdLS and CdCS who had previously participated in research at the university and had agreed to be contacted for future research projects. In addition, some of the participants with CdCS were contacted indirectly through the CdCS Support Group or recruited at support group conferences.

Participants were included at T1 if they met the following criteria:

- Aged between 5 and 19 years.
- Had a confirmed diagnosis of the given syndrome from a professional (paediatrician, clinical geneticist or GP).
- Had no other known genetic abnormality (other than that associated with CdLS and CdCS).
- Lived within reasonable travelling distance of at Cerebra Centre for Neurodevelopmental Disorders, University of Birmingham, UK (approximately 100 miles).

Participants were invited to participate at T2 if they meet the following criteria:

- They met all stated T1 criteria.
- Completed all relevant assessments at T1.
- Agreed to be contacted with information about future research projects.

Two participants were not invited to participate at T2 because they did not meet the inclusion criteria (one did not agree to future contact and one had a queried diagnosis). Two individuals had died prior to the T2 follow up.

### Participants

Thirty-two participants with CdLS and 22 participants with CdCS were invited to take part at T2. Two participants with CdLS declined due to ongoing behavioural challenges, three participants with CdCS did not respond to the invitation to participate and one participant declined to take part. A total of 30 participants with CdLS and 18 participants with CdCS took part at T2. Mean follow-up time for both groups was 81.67 months (CdLS SD = 5.23; CdCS SD = 2.54).

Table 1 describes the characteristics of the participants (CdLS and CdCS) who participated at T1 and T2.

**Table 1:** Participant characteristics at T1 and T2

N		CdLS (30)		CdCS (18)	
		T1	T2	T1	T2
Age (in years)	M (SD) Range	12.24 (3.90) 5–18.96	19.20 (3.91) 12.33–26.54	9.64 (2.72) 5.62–14.56	16.58 (2.81) 12.39–21.69
Gender	% male (N)	46.70 (14)		27.80 (5)	
Speech^a^	% verbal (N)	66.70 (20)	66.7 (20)	72.20 (13)	88.9 (16)
Mobility^a^	% mobile (N)	93.30 (28)	100.00 (30)	94.40 (17)	94.4 (17)
Level of ability^b^	% profound (N)	20.00 (6)	66.70 (20)	11.10 (2)	44.40 (8)
	% severe (N)	53.30 (16)	13.30 (4)	66.70 (12)	50.00 (9)
	% moderate (N)	20.00 (6)	6.70 (2)	11.10 (2)	5.6 (1)
	% mild (N)	6.70 (2)	10.00 (3)	5.60 (1)	0.00 (0)
	% 70+ (N)	0.00 (0)	3.3 (1)	5.60 (1)	0.00 (0)
VABS ABC age equivalence^c^	M (SD) Range	43.83 (28.39) 9.67–98.67	43.64 (35.95) 8.33–144.67	33.64 (17.40) 14.67–77.00	34.91 (18.26) 13.67–78.67
VABS ABC standard score^c^	M (SD)	33.07 (12.38)	27.50 (14.32)	35.17 (17.35)	23.89 (6.06)
Receptive language age equivalence^d^	M (SD)	67.87 (24.48)	81.33 (32.06)	52.76 (23.37)	65.24 (33.37)

^a^Data derived from the demographic questionnaire

^b^Adaptive Behavior Composite classification; derived from the Vineland Adaptive Behavior Scales (Sparrow et al. 1984)

^c^Adaptive Behavior Composite on three domains; derived from the Vineland Adaptive Behavior Scales (Sparrow et al. 1984)

^d^Data derived from the British Picture Vocabulary Scales (Dunn et al. 1997); At T1 12 individuals with CdLS and one individual with CdCS did not pass the test trials on this task. At T2 14 individuals with CdLS and 0 individuals with CdCS did not pass the test trials. Scores are reported based on participants who were able to engage in and complete the task at both time points (CdLS n = 15; CdCS n = 17)

At T1, participants with CdLS were significantly older than participants with CdCS [t (46) = 2.48; *p* = .02]. Participants were comparable on scores of adaptive behaviour [standard scores: t (46)=− .49; *p* = .63; age equivalence: t (46) = 1.37; *p* = .18] and receptive language skills [age equivalence: t (46)=− .16; *p* = .87].

### Measures

#### Background Information

Information regarding gender, chronological age and syndrome diagnosis was gathered from a series of demographic questions.

#### Adaptive Behaviour

The Vineland Adaptive Behavior Scales—Survey form (VABS; Sparrow et al. 1984; the VABS-II and VABS-III were not available when the study began at T1) was conducted over the phone with parents or carers in order to assess each participant’s personal and social adaptive behaviour levels and approximate level of intellectual ability. The measure is a semi-structured interview suitable for use with individuals with or without ID. The VABS is used to evaluate four areas of ability: *communication, daily living socialization* and *motor skills*. Standard scores and age equivalence scores can be calculated for each domain and for the *Adaptive Behavior Composite*. In addition, the *Adaptive Behavior Composite* can be used to determine an approximate severity classification: borderline, mild, moderate, severe and profound. The authors report good validity and high reliability ranging from .83 to .90 for the domains and .94 the *Adaptive Behavior Composite*.

#### Language Ability

The British Picture Vocabulary Scales—2nd Edition (BPVS; Dunn et al. 1997) was used to assess each participant’s receptive language level. This assessment requires the participant to select the picture matching the stimulus word presented orally by the examiner. Split-half reliability and internal consistency are good. Raw, age equivalent and standard scores are provided.

#### ASD Symptomatology

The Social Communication Questionnaire (SCQ; Rutter et al. 2003), an informant-report screening measure, was utilized to collect information about reported potential impairments in reciprocal social interaction, communication, repetitive and stereotyped behaviours. The “current” version (focusing on behaviour from the most recent 3 months) was used at T1 and T2. The measure consists of 40 items each of which requires a *yes* (score of one) or *no* (score of zero) response from a parent/caregiver. The total scores ranges from zero to 39. The authors suggest a cut-off score of 15 for ASDs, which was found to differentiate individuals with autism from those with ID (specificity of .80 and sensitivity of .67). The suggested cut-off for autism is significantly higher at 21 (specificity of .60 and sensitivity of .75; Berument et al. 1999).

The Autism Diagnostic Observation Schedule (ADOS; Lord et al. 2000; the ADOS-2 was not available when the study began at T1) is a semi-structured, standardised observational assessment of communication and social interaction skills, play and imaginative skills and repetitive behaviour, and was used to assess behaviours associated with the autism spectrum. The ADOS is suitable for individuals with a range of developmental abilities, chronological ages and expressive language skills. The use of clear, planned social ‘presses’, provides a good opportunity for the participant to display certain social and communicative behaviours or responses. The presence/absence and nature of these behaviours and responses are recorded. There are four modules (1–4) in the ADOS and module selection is based on the chronological age and level of expressive language of the participant. All modules can be administered quickly (approximately 20–40 min) and each module has its own protocol and scoring algorithm. Algorithm scores were converted to calibrated severity scores using the protocols and normative tables developed by Hus et al. (2014), Gotham et al. (2008), Gotham et al. (2012).

T1 inter-rater reliability for ADOS assessments is reported in *[anonymised for blind review]*. At T2, two authors *[anonymised for blind review]* independently viewed and scored 15 (31.25% of sample) randomly selected videotaped ADOS assessments, blind to the other assessors scores. Kappa coefficients for diagnostic cut off scores were good for the *Communication* domain (.73) and fair to good for the total score (.59) and *Social Interaction* domain (.62). Pearson intra-class correlations also revealed good agreement between the raters on domain and total scores (*Communication* .73, *Social Interaction* .67 and *Total* .60).

#### Mood, Interest and Pleasure

Mood was assessed using The Mood, Interest and Pleasure Questionnaire—Short Version (MIPQ-S; Ross et al. 2008; Ross and Oliver 2003). The MIPQ is an informant based questionnaire used to assess two constructs related to depression: mood and, interest and pleasure. It is designed for use in individuals with a range of intellectual ability including those with severe and profound levels of ID. Informants rate 12 items describing operationally defined observable behaviors to give a total score, a Mood subscale score and an Interest and Pleasure subscale score. The MIPQ is reported to have good internal consistency, test–retest and inter-rater reliability (Ross and Oliver 2003).

#### Repetitive Behaviours

Repetitive behaviours were assessed using The Repetitive Behaviour Questionnaire (RBQ; Moss et al. 2009), an informant measure of repetitive behavior for use in children and adults with a range of ID. The RBQ consists of 19 items that comprise five subscales: stereotyped behavior, compulsive behavior, restricted preferences, insistence on sameness, and repetitive use of language. Informants rate the frequency of each behavior over the preceding month on a five-point Likert scale ranging from ‘never’ to ‘more than once a day’. Inter-rater reliability, test–retest reliability, concurrent validity, content validity and internal consistency are robust (Moss et al. 2009).

### Procedure

Ethical approval for this follow up study was granted by *[anonymised for blind review]*. At T1 all assessments were completed by *[anonymised for blind review]* and at T2 all of the assessments were completed by *[anonymised for blind review]*. Examiners were not blind to the syndrome diagnosis of the participant but the assessor at T2 was blind to T1 assessment scores. The VABS (Sparrow et al. 1984) was conducted with the primary caregiver either by telephone or face to face. Participants were visited at their school/day centre or at home. All ADOS and BPVS assessments were completed in a quiet, distraction free room and were video recorded. ADOS assessments were scored immediately after administration using a combination of live and video recorded observations. Due to a high rate of selective mutism in the CdLS population, ADOS module selection for this study was based on parent/carer reported expressive language level if the participant was selectively mute. Informant questionnaires of mood, interest and pleasure and repetitive behaviour were completed by the participant’s main caregiver.

### Data Analysis

All data were checked for normality. Where data were normally distributed, mixed ANOVAs were conducted with time as the within subjects factor, syndrome group as the between subjects factor and T1 chronological age as a covariate. Where data were not normally distributed, non-parametric analyses were conducted using Mann Whitney U tests to evaluate between group differences at each timepoint, and Wilcoxon tests to evaluate within group change between T1 and T2. Categorical data were evaluated using *χ*^2^ tests to evaluate between group differences and McNemar tests to evaluate change over time. In order to evaluate the extent of change in adaptive behaviour, receptive language skills and autism symptomatology shown by individual participants, the criteria outlined by Jacobson and Truax (1991) to establish the thresholds for reliable significant change were applied. The reliable change index score indicates the threshold at which the degree of change is unlikely (< 5% chance) to be accounted for by measurement unreliability or variability in scores. Calculating reliable change requires the standard deviation score at the baseline from which change is being assessed and an indication of the stability of the measure being used. Cronbach’s alpha reported by the authors of each of the measures conducted in this study was used to indicate measure stability alongside the standard deviation score from the current study total sample.

## Results

Tables 1, 2 report scores on measures of adaptive behaviour, language ability, behaviour and mood at T1 and T2.

**Table 2:** Scores on SCQ, ADOS, MIPQ and RBQ at T1 and T2

N	Subscale/domain		CdLS (30)		CdCS (18)	
			T1	T2	T1	T2
SCQ	Social	M (SD)	5.28 (4.03)	6.28 (3.87)	3.99 (2.54)	2.95 (1.50)
	Communication	M (SD)	4.74 (3.13)	5.47 (2.64)	2.39 (2.00)	2.93 (2.09)
	Repetitive behaviour	M (SD)	2.86 (2.31)	2.99 (1.90)	3.00 (1.68)	3.46 (1.94)
	Total score	M (SD)	13.84 (8.07)	15.91 (7.04)	10.51 (4.12)	10.54 (4.27)
	Autism cut off	% ≥ Cut off	6 (21.43)	6 (21.43)	0 (0.00)	0 (0.00)
	ASD cut off	% ≥ Cut off	11 (39.29)	16 (57.14)	1 (6.67)	3 (20.00)
ADOS	Social affect	M (SD)	6.27 (2.86)	7.20 (2.27)	4.94 (2.21)	4.83 (1.86)
	Repetitive behaviour	M (SD)	4.13 (2.30)	6.03 (2.06)	4.61 (2.45)	7.17 (2.60)
	Total score	M (SD)	13.00 (7.53)	6.80 (2.11)	11.33 (6.32)	5.33 (1.94)
	Autism cut off	n ≥ Cut off (%)	17 (56.67)	24 (80.00)	6 (33.33)	9 (50.00)
	ASD cut off	n ≥ Cut off (%)	20 (66.67)	28 (93.30)	12 (66.67)	14 (77.78)
MIPQ	Mood	M (SD)	19.80 (3.04)	19.80 (3.04)	20.06 (2.51)	20.06 (2.51)
	Interest & pleasure	M (SD)	15.55 (3.88)	15.55 (3.88)	15.06 (4.52)	15.06 (4.52)
	Total score	M (SD)	35.35 (5.86)	35.35 (5.86)	35.12 (6.66)	35.12 (6.66)
RBQ	Stereotyped behaviour	Median Range	4.00 0.00–12.00	5.00 0.00–12.00	4.50 0.00–12.00	5.00 0.00–12.00
	Compulsive behaviour	Median Range	5.00 0.00–18.29	4.00 0.00–28.00	2.00 0.00–16.00	4.00 0.00–19.00
	Insistence on sameness	Median Range	0.00 0.00–8.00	3.00 0.00–8.00	1.00 0.00–8.00	1.00 0.00–8.00
	Restricted interests	Median Range	2.50 0.00–10.00	5.00 0.00–8.00	2.00 0.00–10.00	7.00 0.00–8.00
	Repetitive language	Median Range	4.00 0.00–8.00	4.00 0.00–12.00	2.00 0.00–11.00	4.00 0.00–12.00
	Total score	Median Range	15.00 0.00–48.00	17.00 3.00–56.00	14.50 0.00–46.00	21.00 7.00–47.00

### Adaptive Behaviour

Repeated measures ANOVA with T1 chronological age as a covariate revealed a significant main effect of time [F (1, 45) = 55.45; *p* < .001] with both groups showing a decline in Adaptive Behaviour Composite standard scores between T1 and T2. This was not the case for Adaptive Behaviour Composite age equivalent scores, which remained relatively stable in both groups over time. No main effects of group or group by time interactions were identified for adaptive behaviour standard or age equivalence scores. Domain level scores revealed a similar pattern of overall decline in standard scores, with significant main effects of time observed for standard scores on the Communication [F (1, 45) = 74.68; *p* < .001], Socialization [F (1, 45) = 35.16; *p* < .001] and Daily Living Skills [F (1, 45) = 20.11; *p* < .001]. No significant main effects of group, or group by time interactions, were identified for age equivalence domain level scores.

### Language Ability

Twelve participants with CdLS and one participant with CdCS did not successfully complete the test trials of the BPVS at T1. A further three participants with CdLS failed to complete these test trials at T2. Consequently, analysis of BPVS scores includes only those individuals who were able to complete the task at both time points. A repeated measures ANOVA with T1 chronological age as a covariate revealed a significant main effect of time [F (1, 29) = 15.21; *p* = .001], with both groups showing significant improvement in BPVS age equivalence scores between T1 and T2. The group by time interaction approached significance [F (1, 29) = 3.80; *p* = .06] with the CdLS group making more significant improvements in receptive language skills relative to the CdCS group over time. A similar pattern of results was evident for BPVS raw scores. Analyses were repeated, including only participants who completed the assessment at T1. This did not change the statistical significance of the analyses.

### ASD Symptomatology

There were no significant changes in either syndrome group in the proportion of participants meeting cut off scores for ASD or autism on the SCQ. On the ADOS, there was a significant increase in the proportion of participants with CdLS who scored above the cut off for autism (*p* = .04) and ASD (*p* = .008) between T1 and T2. No significant differences were observed within the CdCS group.

Repeated measures ANCOVA with group as the between subjects factor, time as the within subjects factor and T1 chronological age as a covariate identified a significant time × group interaction on the reciprocal social interaction subscale of the SCQ [F (1, 41) = 5.61; *p* = .02]; the CdLS group scored significantly higher than the CdCS group on this subscale at T2 but not at T1. No significant main effects of time or group or interactions were identified for the Total, Communication or Repetitive Behaviour scores on the SCQ. Significant main effects of group on the ADOS Social Affect Severity score [F (1, 45) = 9.26; *p* = .004] and the ADOS Total Severity score [F (1, 45) = 5.03; *p* = .03] were identified. In both cases, the CdLS group scored significantly higher than the CdCS group at both time points. There was no significant main effect of time and no time by syndrome interaction on either score. Non-parametric between group contrasts revealed no significant difference on Stereotyped and Repetitive Behaviour Severity scores at T1 (U = 240.00; *p* = .51). A significant group difference was however observed at T2 (U = 159.00; *p* = .02) with the CdCS group scoring significantly higher than the CdLS group. Non-parametric within group contrasts between T1 and T2 identified a significant increase in scores over time on the Stereotyped and Repetitive Behavior Severity score in both groups (CdLS: Z = − 3.37, *p* = .001; CdCS: Z = − 2.56; *p*.01).The findings from these non-parametric tests indicate a group by time interaction, although this cannot be fully evaluated using non-parametric methods, which were used due to the skewed distribution of the data.

### Mood, Interest and Pleasure

A significant group by time interaction was identified on both the total MIPQ score [F (1, 36) = 6.18; *p* = .02] and on the interest and pleasure subscale [F (1, 36) = 7.02; *p* = .01]. Pair-wise post hoc contrasts showed a significant increase in total MIPQ score and interest and pleasure subscale scores over time in the CdCS group (*p* = .01). No significant change over time was observed in the CdLS group who scored significantly lower on the interest and pleasure subscale than the CdCS group at T2 but not at T1 (*p* = .04). No significant main effects or group by time interaction was identified for the mood subscale.

### Repetitive Behaviour

Non-parametric between group comparisons identified no group differences at T1 or T2 on the total RBQ score or on any RBQ subscale score. No significant change over time was identified on the RBQ (total or subscale scores) in either syndrome group.

### Patterns of Change for Individuals over Time

In order to examine the extent of change shown by individual participants, the proportion of individuals who made reliable change in specific areas between T1 and T2 was calculated. Table 3 shows the pattern of individual change on measures of adaptive behaviour, language and behavioural characteristics. Since scores on the Repetitive Behaviour Questionnaire were not normally distributed this measure was not included within this analysis.

**Table 3:** Reliable change outcome on measures of ASD, adaptive behaviour, language skills and mood

	Age at T1 (years)	VABS ABC T1 (AE months)	T1–T2 change scores					
			ADOS total severity score		SCQ total score	VABS ABC (AE)	BPVS (AE)	MIPQ total score
CdLS								
1	12.46	20.33	0		0	0	0	0
2	13.09	20.67	0		0	0	0	+
3	6.83	59.33	−		0	+	+	0
4	14.73	10.00	0		0	0	0	0
5	8.04	12.67	0		+	0	0	0
6	8.15	15.33	0		−	0	0	0
7	6.57	30.00	0		−	+	0	−
8	10.30	32.30	0		0	0	0	0
9	18.96	98.00	0		0	−	0	+
10	13.87	52.00	0		0	+	+	0
11	17.97	41.33	0		0	0	0	−
12	13.49	36.00	0		0	0	+	0
13	9.80	15.67	0		0	0	0	−
14	5.76	14.00	0		0	0	0	+
15	8.86	9.67	0		0	0	0	0
16	10.72	10.33	0		0	0	0	−
17	18.69	77.67	0		0	−	0	−
18	12.84	15.00	0		0	0	0	0
19	11.01	53.00	0		−	0	0	0
20	5.00	30.00	0		0	0	0	0
21	11.56	45.00	−		0	−	0	−
22	10.73	75.60	0		0	+	+	0
23	10.14	48.60	−		−	0	0	0
24	12.80	39.30	0		0	−	0	0
25	16.52	90.60	−		0	+	+	0
26	16.87	63.30	0		0	−	0	+
27	13.83	52.30	0		0	0	0	−
28	17.55	98.67	0		0	−	0	0
29	15.85	96.00	0		0	+	+	+
30	14.28	52.30	0		0	0	0	0
CdCS								
1	13.83	15.67	0		0	0	+	0
2	13.15	31.67	0		0	0	0	+
3	7.70	21.33	0		0	+	0	+
4	8.52	25.67	0		0	0	−	+
5	6.82	24.00	0		0	0	0	+
6	10.28	34.67	0		0	0	0	0
7	12.55	14.67	0		0	+	0	+
8	7.43	58.00	0		0	0	+	0
9	9.97	25.67	0		0	0	0	+
10	14.56	32.00	0		0	0	0	0
11	9.20	26.60	0		0	0	0	0
12	9.80	49.30	−		−	−	0	0
13	10.49	45.30	0		0	0	0	+
14	12.02	59.60	0		0	+	0	0
15	8.63	15.00	0		0	0	0	−
16	5.62	24.30	0		0	0	0	+
17	5.73	77.00	0		0	−	+	0
18	7.26	25.00	0		0	−	0	0

− Indicates a clinically significant decline in ability (for SCQ and ADOS this is inferred by an increase in scores. For all other measures this is inferred by a decrease in score)

+ Indicates a clinically significant improvement in ability (for SCQ and ADOS this is inferred by a decrease in scores. For all other measures this is inferred by an increase in score)

0 Indicates no clinically significant change

Just over half of the CdLS sample (n = 16) showed a significant decline in performance in one or more areas evaluated. Of these individuals, five participants also showed significant improvements in at least one area. A further seven participants showed only improvements (in one of more areas) over time, with no significant areas of decline. Seven participants remained stable across all areas between T1 and T2.

Only five individuals with CdCS showed a significant decline in performance in one or more areas evaluated, two of which also showed improvements in at least one area. Ten participants showed significant improvements in one or more areas, with no significant decline in scores on any measure. Three participants remained entirely stable across all areas between T1 and T2.

Visual inspection of the areas in which reliable change (improvement or decline) in performance were identified revealed the following: In relation to receptive language skills age equivalence, six individuals with CdLS and three with CdCS showed a reliable improvement between T1 and T2. One participant with CdCS showed a decline in these skills. Six participants with CdLS also showed a reliable decline in VABS age equivalence score, while five participants showed a reliable increase in scores. Only three participants with CdCS showed a reliable decline in VABS age equivalence score and a further three participants showed a reliable increase on this score.

Seven participants with CdLS showed a significant decline in performance on either total ADOS severity score and/or total SCQ score (as indicated by increased severity or total scores) compared to only one participant with CdCS. Only one participant with CdLS made improvements in this area between T1 and T2. In relation to scores on the MIPQ, seven individuals with CdLS compared to only one individual with CdCS showed a significant decline in total MIPQ score between T1 and T2. Five individuals with CdLS and eight individuals with CdCS showed a reliable increase in total MIPQ scores. The findings demonstrate that the nature and direction of change within and between participant groups is highly variable at the individual level.

## Discussion

The purpose of this study was to evaluate the stability of adaptive behaviour, language skills and behavioural characteristics including ASD symptomatology, mood and repetitive behaviour in individuals with CdLS and a contrast group of individuals with CdCS over a 7 year follow up period. This is the first longitudinal study of individuals with CdLS over a follow up period of this length and is the first longitudinal follow up study to consider broader aspects of change including adaptive behaviour and receptive language skills in these groups.

The findings demonstrate broad stability or improvement in adaptive behaviour and language skills in both syndrome groups over time, alongside syndrome specific profiles of change (improvement and decline) among behavioural variables. Inspection of reliable change scores for individual participants indicate heterogeneity within both groups with regard to the nature of improvements and decline over time.

### Adaptive Behaviour and Language

Both groups demonstrated a significant decline in standard scores in all areas of adaptive behaviour alongside stability of age equivalence scores over time. This pattern of results indicates that adaptive behaviour skills are likely to have plateaued between T1 and T2 in both groups. In contrast to these findings, both groups made significant gains in receptive language skills over time. Interestingly, the number of individuals with CdLS who were unable to perform on the receptive language task increased between T1 and T2. It is not clear whether this change in ability to perform was due to genuine loss of skills or was related to other test related factors such as demand avoidance or increased performance anxiety. Including those individuals who were not able to perform on the task (by allocating them the lowest possible raw and age equivalence scores) did not change the outcome of statistical analyses. While decline in specific aspects of cognitive function (specifically executive function skills) with increasing age have previously been described within the literature (Reid et al. 2017), this is the first study to consider changes in broader areas of language, cognitive and adaptive functioning. The fact that adaptive behaviour skills are relatively stable over time and that receptive language skills improve significantly in both syndrome groups suggests that any changes observed in individuals with CdLS with age are unlikely to be indicative of a global decline in ability.

### ASD Symptomatology and Repetitive Behaviour

According to scores on the SCQ, the proportion of individuals meeting the cut off for autism and ASD remained stable between T1 and T2. Domain scores, however, indicated that individuals with CdLS showed increased severity in social impairments over time. This pattern of results was not identified in the CdCS group.

The current study findings from the SCQ are broadly consistent with previous reports indicating greater severity of social impairments over time. Descriptive studies of individuals with CdLS describe increased social withdrawal and social isolation in older individuals with the syndrome (Sarimski 1997; Kline et al. 2007a). These findings have been confirmed by cross sectional studies that have demonstrated more severe ASD symptomatology in individuals with CdLS aged 15 years and older (Nelson et al. 2014). While a longitudinal study conducted over a 2.5 year period demonstrated stability in the severity of ASD symptomatology over time (Cochran et al. 2015). The pattern of increased severity of ASD related social impairments over time directly contrasts with patterns reported in the idiopathic ASD population in which symptoms are considered to improve over time, despite stability in diagnoses (see Magiati et al. 2014, for a review of 25 studies).

Outcome on the ADOS indicated a somewhat different pattern of results to that identified by the SCQ. According to ADOS algorithm scores, there was a significant increase in the proportion of individuals with CdLS who met cut off for autism and ASD between T1 and T2. Evaluation of calibrated severity scores indicated that social affect was more impaired in individuals with CdLS at both T1 and T2 compared to the CdCS group. Both groups also demonstrated significantly increased severity of repetitive behaviour over time; however, this change was more prominent in those with CdCS. This pattern of findings relating to repetitive behaviour was not replicated in the RBQ.

The lack of consistency between the SCQ and ADOS measures hinders interpretation of findings and calls into question the concurrent validity of these two assessments within these two populations. This requires further investigation. However, the discrepancy may also be accounted for by differences between the two measures with regard to the way in which constructs of ASD symptomatology are conceptualised and categorised. For example, the SCQ distinguishes between social and communication skills across two domains while the ADOS calibrated severity scores combine both social and communication skills into a single domain. According to results from the SCQ, the changes observed in individuals with CdLS are very specific to social abilities and this cannot be evaluated specifically by using the ADOS severity scores. The need for a more refined approach in order to understand fully the nature of ASD symptomatology in individuals with genetic syndromes is demonstrated widely across the behavioural phenotypes literature (e.g., Moss et al. 2008; Richards et al. 2016; McDuffie et al. 2015; see; Jeste and Geschwind 2014, for a review). The degree of specificity required in these populations may not be possible to identify by relying upon the calibrated social and communication ADOS algorithm scores.

### Mood, Interest and Pleasure

The findings in relation to mood, interest and pleasure are somewhat inconsistent with previous research in CdLS and suggest no clear change in this area between T1 and T2. Previous studies of individuals with CdLS indicate that changes in mood and behaviour are most likely to become apparent in late adolescence or early adulthood (Basile et al. 2007; Berney et al. 1999; Kline et al. 2007a) with the period between 19 and 22 years of age being a critical point for experiencing significantly lower mood, interest and pleasure (Nelson et al. 2014). The mean age of participants with CdLS at T2 in the current study was 19.2 years. It is therefore possible that our observations were conducted on the threshold of this critical change period for our sample and therefore changes may not have been detectable at this time point. This is particularly likely given emerging evidence that points towards a steady, incremental change during this critical age period (Groves et al. in review). Interestingly, at the group level, individuals with CdCS showed a significant improvement in mood, interest and pleasure over time.

### Patterns of Individual Change

Given the extent of genotypic and phenotypic heterogeneity that has previously been documented in individuals with CdLS (Jackson et al. 1993; Ansari et al. 2014), it was considered important to look more closely at patterns of change in individual participants. Based on visual inspection of the data, and at the broadest level of evaluation, individuals with CdLS were more likely to show a decline in at least one area of assessment over the follow up period compared to the CdCS group. Within the CdLS sample, the pattern of decline and improvement on individual assessments was highly variable across participants, suggesting that group trends should not be considered to reliably predict temporal change for individuals clinically. Recent evidence suggests that variability with regard to the nature of changes with age in CdLS might be associated with genetic variability (Moss et al. 2017). However, further investigation of the specific participant characteristics that mediate this variability is required and will be important for ensuring early identification of those individuals most at risk for changes with age.

### Study Limitations

There are several study limitations that need to be considered when interpreting the findings from this study. First, genetic information was not available for this cohort, meaning that any relationships between genetic variability and patterns of change over time remain unassessed. Second, although small sample sizes are a common limitation of work with rare genetic syndromes, and despite the fact that the follow-up rate for the CdLS group in this study is strong, the N of 30 may not provide the power to detect all temporal changes for the group. Also, whilst the CdCS group is well matched to the CdLS group on key variables, the attrition rate in this comparison group was greater, and the use of a single comparison group with its own behavioural phenotype introduces the possibility that specific characteristics of the comparison group mask or accentuate apparent behavioural features/changes in the main group under study. Furthermore, future research assessing change in cohorts of specific ages may be important. The ages of participants in this study at the point of initial assessment varied from 5 to 19 years, and thus the period of change under investigation varied across participants (from 5–12 years to 19–27 years). Notably, the current study does not allow detailed specific consideration of any possible changes in the 19–22 age-group, despite previous indications that this may be a time of significant change in mood (Nelson et al. 2014). The potentially interacting roles of psychosocial factors (e.g., adjustment to peers moving away; changes in educational support) and neurobiological changes at these points in life have yet to be elucidated. In addition, development into middle and later adulthood should form a focus of future work.

### Summary and Conclusions

A 7 year follow-up study of a broad range of behavioural/psychological characteristics was conducted for a group of 30 individuals with CdLS, and a comparison group of 18 individuals with CdCS. Results indicated a complex pattern of change and stability over time, at both group and individual levels. In line with previous cross-sectional studies, the current data suggest an increase in autism spectrum symptomatology for the CdLS group, although previous suggestions of declining mood over time were not borne out by the current study. Stable adaptive ability and improving receptive language suggest that any changes over time are not likely to be the result of an overall decline in ability. Analysis of individuals’ data indicates considerable heterogeneity in temporal change; thus, the observed trends cannot be considered predictive of outcomes for individuals. The manner in which heterogeneity in behavioural/psychological development may relate to genetic, medical and neurophysiological heterogeneity within the syndrome groups remains to be established by future research.
