# Correction to: Age-related Behavioural Change in Cornelia de Lange and Cri du Chat Syndromes: A Seven Year Follow-up Study

**DOI:** 10.1007/s10803-019-04031-y

**Published:** 2019-05-27

**Authors:** Lisa Cochran, Alice Welham, Chris Oliver, Adam Arshad, Joanna F. Moss

**Affiliations:** 1 Cerebra Centre for Neurodevelopmental Disorders, School of Psychology, University of Birmingham, Birmingham, UK; 2 Institute of Cognitive Neuroscience, University College London, London, UK; 3 University of Leicester, Leicester, UK; 4 College of Medical and Dental Sciences, University of Birmingham, Birmingham, UK

## Correction to: Journal of Autism and Developmental Disorders 10.1007/s10803-019-03966-6

The original version of this article unfortunately published with the incorrect text “details removed for blind review” instead of “Cerebra Centre for Neurodevelopmental Disorders, University of Birmingham, UK”. In addition, there are few typos in Tables 2 and 3 which are corrected only in this erratum.

**Table 2:** Scores on SCQ, ADOS, MIPQ and RBQ at T1 and T2

N	Subscale/domain		CdLS (30)		CdCS (18)	
			T1	T2	T1	T2
SCQ	Social	M (SD)	5.28 (4.03)	6.28 (3.87)	3.99 (2.54)	2.95 (1.50)
	Communication	M (SD)	4.74 (3.13)	5.47 (2.64)	2.39 (2.00)	2.93 (2.09)
	Repetitive behaviour	M (SD)	2.86 (2.31)	2.99 (1.90)	3.00 (1.68)	3.46 (1.94)
	Total score	M (SD)	13.84 (8.07)	15.91 (7.04)	10.51 (4.12)	10.54 (4.27)
	Autism cut off	% ≥ Cut off	6 (21.43)	6 (21.43)	0 (0.00)	0 (0.00)
	ASD cut off	% ≥ Cut off	11 (39.29)	16 (57.14)	1 (6.67)	3 (20.00)
ADOS	Social affect	M (SD)	6.27 (2.86)	7.20 (2.27)	4.94 (2.21)	4.83 (1.86)
	Repetitive behaviour	M (SD)	4.13 (2.30)	6.03 (2.06)	4.61 (2.45)	7.17 (2.60)
	Total score	M (SD)	5.40 (2.74)	6.80 (2.11)	4.56 (1.89)	5.33 (1.94)
	Autism cut off	n ≥ Cut off (%)	17 (56.67)	24 (80.00)	6 (33.33)	9 (50.00)
	ASD cut off	n ≥ Cut off (%)	20 (66.67)	28 (93.30)	12 (66.67)	14 (77.78)
MIPQ	Mood	M (SD)	19.80 (3.04)	19.80 (3.04)	20.06 (2.51)	20.06 (2.51)
	Interest & pleasure	M (SD)	15.55 (3.88)	15.55 (3.88)	15.06 (4.52)	15.06 (4.52)
	Total score	M (SD)	35.35 (5.86)	35.35 (5.86)	35.12 (6.66)	35.12 (6.66)
RBQ	Stereotyped behaviour	Median Range	4.00 0.00–12.00	5.00 0.00–12.00	4.50 0.00–12.00	5.00 0.00–12.00
	Compulsive behaviour	Median Range	5.00 0.00–18.29	4.00 0.00–28.00	2.00 0.00–16.00	4.00 0.00–19.00
	Insistence on sameness	Median Range	0.00 0.00–8.00	3.00 0.00–8.00	1.00 0.00–8.00	1.00 0.00–8.00
	Restricted interests	Median Range	2.50 0.00–10.00	5.00 0.00–8.00	2.00 0.00–10.00	7.00 0.00–8.00
	Repetitive language	Median Range	4.00 0.00–8.00	4.00 0.00–12.00	2.00 0.00–11.00	4.00 0.00–12.00
	Total score	Median Range	15.00 0.00–48.00	17.00 3.00–56.00	14.50 0.00–46.00	21.00 7.00–47.00

**Table 3:** Reliable change outcome on measures of ASD, adaptive behaviour, language skills and mood

	Age at T1 (years)	VABS ABC T1 (AE months)		T1–T2 change scores				
				ADOS total severity score	SCQ total score	VABS ABC (AE)	BPVS (AE)	MIPQ total score
CdLS								
1	12.46	20.33		0	0	0	0	0
2	13.09	20.67		0	0	0	0	+
3	6.83	59.33		−	0	+	+	0
4	14.73	10.00		0	0	0	0	0
5	8.04	12.67		0	+	0	0	0
6	8.15	15.33		0	−	0	0	0
7	6.57	30.00		0	−	+	0	−
8	10.30	32.30		0	0	0	0	0
9	18.96	98.00		0	0	−	0	+
10	13.87	52.00		0	0	+	+	0
11	17.97	41.33		0	0	0	0	−
12	13.49	36.00		0	0	0	+	0
13	9.80	15.67		0	0	0	0	−
14	5.76	14.00		0	0	0	0	+
15	8.86	9.67		0	0	0	0	0
16	10.72	10.33		0	0	0	0	−
17	18.69	77.67		0	0	−	0	−
18	12.84	15.00		0	0	0	0	0
19	11.01	53.00		0	−	0	0	0
20	5.00	30.00		0	0	0	0	0
21	11.56	45.00		−	0	−	0	−
22	10.73	75.60		0	0	+	+	0
23	10.14	48.60		−	−	0	0	0
24	12.80	39.30		0	0	−	0	0
25	16.52	90.60		−	0	+	+	0
26	16.87	63.30		0	0	−	0	+
27	13.83	52.30		0	0	0	0	−
28	17.55	98.67		0	0	−	0	0
29	15.85	96.00		0	0	+	+	+
30	14.28	52.30		0	0	0	0	0
CdCS								
1	13.83	15.67		0	0	0	+	0
2	13.15	31.67		0	0	0	0	+
3	7.70	21.33		0	0	+	0	+
4	8.52	25.67		0	0	0	−	+
5	6.82	24.00		0	0	0	0	+
6	10.28	34.67		0	0	0	0	0
7	12.55	14.67		0	0	+	0	+
8	7.43	58.00		0	0	0	+	0
9	9.97	25.67		0	0	0	0	+
10	14.56	32.00		0	0	0	0	0
11	9.20	26.60		0	0	0	0	0
12	9.80	49.30		−	−	−	0	0
13	10.49	45.30		0	0	0	0	+
14	12.02	59.60		0	0	+	0	0
15	8.63	15.00		0	0	0	0	−
16	5.62	24.30		0	0	0	0	+
17	5.73	77.00		0	0	−	+	0
18	7.26	25.00		0	0	−	0	0

− Indicates a clinically significant decline in ability (for SCQ and ADOS this is inferred by an increase in scores. For all other measures this is inferred by a decrease in score)

+ Indicates a clinically significant improvement in ability (for SCQ and ADOS this is inferred by a decrease in scores. For all other measures this is inferred by an increase in score)

0 Indicates no clinically significant change

The original article has been corrected.

